# Association of prehospital airway management technique with survival outcomes of out-of-hospital cardiac arrest patients

**DOI:** 10.1371/journal.pone.0269599

**Published:** 2022-06-06

**Authors:** Eujene Jung, Young Sun Ro, Hyun Ho Ryu, Sang Do Shin

**Affiliations:** 1 Department of Emergency Medicine, Chonnam National University Hospital, Gwangju, Republic of Korea; 2 Department of Emergency Medicine, Seoul National University College of Medicine, Seoul, Republic of Korea; 3 Chonnam National University College of Medicine, Gwangju, Republic of Korea; Stony Brook University Renaissance School of Medicine, UNITED STATES

## Abstract

**Introduction:**

Despite numerous studies on airway management in out-of-hospital cardiac arrest (OHCA) patients, the choice of prehospital airway management technique remains controversial. Our study aimed to investigate the association between prehospital advanced airway management and survival outcomes according to a transport time interval (TTI) using nationwide OHCA registry database in Korea.

**Methods:**

The inclusion criteria were patients with OHCA aged over 18 years old with a presumed cardiac etiology between January 2015 and December 2018. The primary outcome was survival to hospital discharge. The main exposure was the prehospital airway management technique performed by the emergency medical technicians (EMTs), classified as bag-valve mask (BVM), supraglottic airway (SGA), or endotracheal intubation (ETI).We performed multivariable logistic regression analysis and interaction analysis between the type of airway management and TTI for adjusted odds ratios (aORs) and 95% confidence intervals (CIs).

**Results:**

Of a total of 70,530 eligible OHCA patients, 26,547 (37.6%), 38,391 (54.4%), and 5,592 (7.9%) were managed with BVM, SGA, ETI, respectively. Patients in the SGA and ETI groups had a higher odds of survival to discharge than BVM groups (aOR, 1.11 (1.05–1.16) and 1.13 (1.05–1.23)). And the rates of survival to discharge with SGA and ETI were significantly higher in groups with TTI more than 8 minutes (1.17 (1.08–1.27) and 1.38 (1.20–1.59)).

**Conclusion:**

The survival to discharge was significantly higher among patients who received ETI and SGA than in those who received BVM. The transport time interval influenced the effect of prehospital airway management on the clinical outcomes after OHCA.

## Introduction

Out-of-hospital cardiac arrest (OHCA) is a significant public health burden due to its high morbidity and low rates of survival. It was reported that the survival to discharge rate was lower in Asia (2%) than in Europe (9%) and North America (6%) [1–4].

There are limited proven treatments that can improve the survival outcomes of OHCA patients at the prehospital stage [5]. Prehospital airway management including bag-valve mask (BVM), supraglottic airway (SGA), and endotracheal intubation (ETI) is an essential components of bundle care of OHCA for improving clinical outcomes [6]. However, several studies have shown various contradictory results regarding the effectiveness of airway management technique in OHCA patients. In the UK randomized controlled trial (RCT), SGA did not result in a favorable functional outcome compared to ETI [2], the PART trial showed that laryngeal tube showed better clinical outcomes compared to ETI [4], and the CAAM trial showed slightly higher 28-day favorable neurological outcomes than ETI [3].

ETI plays an important role in oxygen ventilation; however, its use in patients with OHCA is controversial. Several studies have reported significant rates of misplacement of the tube, insertion failure, iatrogenic hyperventilation, and chest compression interruptions during ETI [7, 8]. SGA insertion can be performed rapidly and requires brief training compared to that required for ETI; however, SGA might lead to misplacement of the tube, airway trauma, and aspiration of gastric content compared with ETI [9, 10]. Some studies reported decreased carotid artery blood flow after the insertion of SGA in a porcine model [11]. Although BVM is simple and practical to perform, it has some disadvantages, including increased risk of gastric regurgitation, pulmonary aspiration, and difficultly in providing adequate ventilation due to the difficulty in sealing the BVM. Advanced airway management (AAM) can reduce complications commonly associated with BVM ventilation during transport. Therefore, pre-hospital AAM may improve survival outcomes for patients who require extended transport time interval (TTI) [12].

Despite numerous studies on airway management in OHCA patients, the choice of prehospital airway management technique in OHCA remains controversial. In this study, we investigated whether the type of prehospital airway management technique is associated with survival outcomes in patients with OHCA, using a nationwide OHCA registry. We further investigated whether the effect of airway management in patients with OHCA is affected by the duration of TTI.

## Methods

This study was approved by the institutional review board of the Chonnam national university hospital, and the need for informed consent was waived (2020–09018).

### Study design

This was a retrospective observational study using the nationwide OHCA registry database in Korea. This study was approved by the institutional review board of the study hospital, and the need for informed consent was waived.

### Data source

The nationwide OHCA registry was first created in 2006 in collaboration with the National Fire Agency and Korea Centers for Disease Control and Prevention (CDC), to improve the survival outcomes of cardiovascular disease in Korea. Data were collected from the EMS run sheets for information about basic ambulance operation, from the Emergency medical service (EMS) cardiac arrest registry for Utstein factors, and from the national OHCA registry for hospital care and survival outcomes, which were reviewed and extracted from the hospital medical records by the Korea CDC. EMS providers record the EMS run sheets and EMS cardiac registry for every case of OHCA after transporting the patients to an emergency department (ED). Ambulance run sheets are electronically stored in each provincial EMS headquarter, which is operated by the fire department. The EMS records include the following data: age, sex, place of event, witness to the event, CPR administered by the bystander, initial ECG rhythm, defibrillation performed by the bystander, time before call for ambulance and hospital arrival and the procedures and care provided by emergency medical technicians (EMT). Trained medical record reviewers visited the study hospitals (approximately 900 hospitals) and reviewed the medical records for Utstein factors and outcomes such as survival to admission, survival to discharge, and neurological recovery. A quality management committee (QMC) trained all the medical record reviewers prior to the start of the project and provided not only a standard manual for data collection but also monthly feedback to the reviewers. The reviewers consulted an emergency medicine physician from the QMC for clarification when they were unable to define a coding element. Another source of data, the registry recorded by medical control dispatchers in the dispatcher center, was used. The dispatchers recorded all the medical controls and pre-arrival instructions in a designated registry [13].

### Study setting

The Korean EMS system is a single-tiered, government-based system operated by 16 provincial headquarters of the National Fire Department, covering a population of approximately 50 million. There are approximately 1,400 ambulance stations nationwide.

EMTs in Korea are classified into level-1 and level-2 EMTs (comparable to EMT-intermediate and EMT-basic in the United States, respectively). According to the Emergency Medical Service Act, level-1 EMTs should have graduated from an EMT school of a university or college and should have passed a national certification examination comprising written and practical skill tests. The curriculum of the EMT school for advanced airway management should include 6 courses and 147 hours of education, with lectures and skill laboratories. After passing the national certification examination, certified level-1 EMTs can apply for the Fire Service Academy during recruitment. Only level-1 EMTs can perform AAM under direct medical control. All ambulance crew can perform CPR at a scene and during transport. The current EMS CPR protocol calls for EMTs to perform CPR, using AED every 2 minutes, for at least 5 minutes on scene. EMTs cannot declare death in the field unless there are signs of irreversible death (rigor mortis, dependent lividity, decapitation, trans-section and decomposition) and it is confirmed by direct medical control. After delivery of more than 5 minutes of chest compressions, EMTs should transport the OHCA victim as soon as possible to the nearest emergency department (ED) while continuing CPR during transport. The airway management technique is to be performed only by level-1 EMTs on-scene under the direct or indirect medical control by a medical director. The choice of airway management technique is relatively freely selected according to the advice of the medical director, the transport time interval, and the skill of the EMT. Ambulance personnel cannot declare death at the scene or terminate CPR until the return of spontaneous circulation (ROSC). Thus, all patients with OHCA are transported to an ED.

In Korea, all EDs are designated as level 1, 2, or 3 by the government, with the designation level based on the availability of human resources, intensive care units, instruments, and equipment available at each ED. There are 460 EDs that are categorized into three levels according to the capacity and resources such as equipment, staffing, and size of the ED. Level-1 EDs (n = 20) provide 24-hour/365-day emergency care by emergency specialists, level-2 EDs (n = 110) include emergency physicians, and level-3 EDs (n = 310) can include general physicians. All EDs generally perform acute cardiac management and post-resuscitation care in accordance with the international standard guidelines such as the 2015 American Heart Association guidelines [14].

### Study population

Data between January 2015 and December 2018 were extracted. The inclusion criteria were patients with OHCA aged >18 years old with a presumed cardiac etiology. The etiology of cardiac arrest was identified by medical record reviews, and cases with primary non-cardiac etiology were excluded. We assumed the presence of a primary cardiac etiology if there was no description of a definite non-cardiac etiology such as trauma, poisoning, drowning, hanging, exsanguination, burns, or asphyxia in the medical records.

Patients who did not receive any CPR at the EMS, those treated by level-2 EMTs, and those not treated using one of the three (BVM, SGA, and ETI) airway management techniques were excluded. Patients were also excluded if the information about the method of prehospital airway management or clinical outcomes at discharge could not be obtained.

### Main outcomes

The primary outcome was survival to hospital discharge. The secondary outcome was a neurologically favorable survival to hospital discharge, defined as a Glasgow–Pittsburgh cerebral performance category (CPC) of 1 or 2 [15]. The CPC score was determined by the medical record reviewers based on the discharge summary and documentation in the medical records.

### Variables and measurements

The main exposure variable was the prehospital airway management technique performed by the EMTs, classified as BVM, SGA, or ETI. The selection of the airway management technique completely depended on the preference of the level 1 EMT at the scene.

Patient characteristics were obtained from the national OHCA registry. We collected information about the age, sex, pre-existing disease (hypertension, diabetes mellitus, and heart disease), location of arrest (public or private), witnessed status, CPR administered by the bystander, ECG results, and level of ED. We also collected EMS information about the duration of time from the call to ambulance arrival at the scene (response time interval), from ambulance arrival to departure from the scene (scene time interval), and from departure from the scene to arrival at the hospital (transport time interval). The use of an electronic database prevented skipped entries and missing data. For quality assurance, monthly data quality management programs were held by the Korea CDC.

### Statistical analysis

We compared the patient demographics, characteristics of the cardiac arrest, EMS time intervals and procedures, and study outcomes according to the type of prehospital airway management technique received (BVM, SGA, and ETI) using the Chi-square test for categorical variables and the Wilcoxon rank-sum test for continuous variables.

Univariable and multivariable logistic regression analyses were performed to estimate the effect sizes of different types of prehospital airway management techniques on survival to discharge and good neurological outcome. Crude and adjusted odds ratios (ORs) with 95% confidence intervals (CIs) were calculated. Finally, the interaction between the type of prehospital airway management technique and TTI was also analyzed.

All statistical analyses were performed using SAS version 9.4 (SAS Institute Inc., Cary, NC, USA). All p-values were two-tailed, and p < 0.05 was considered statistically significant.

## Results

### Demographic findings

**Fig 1:**
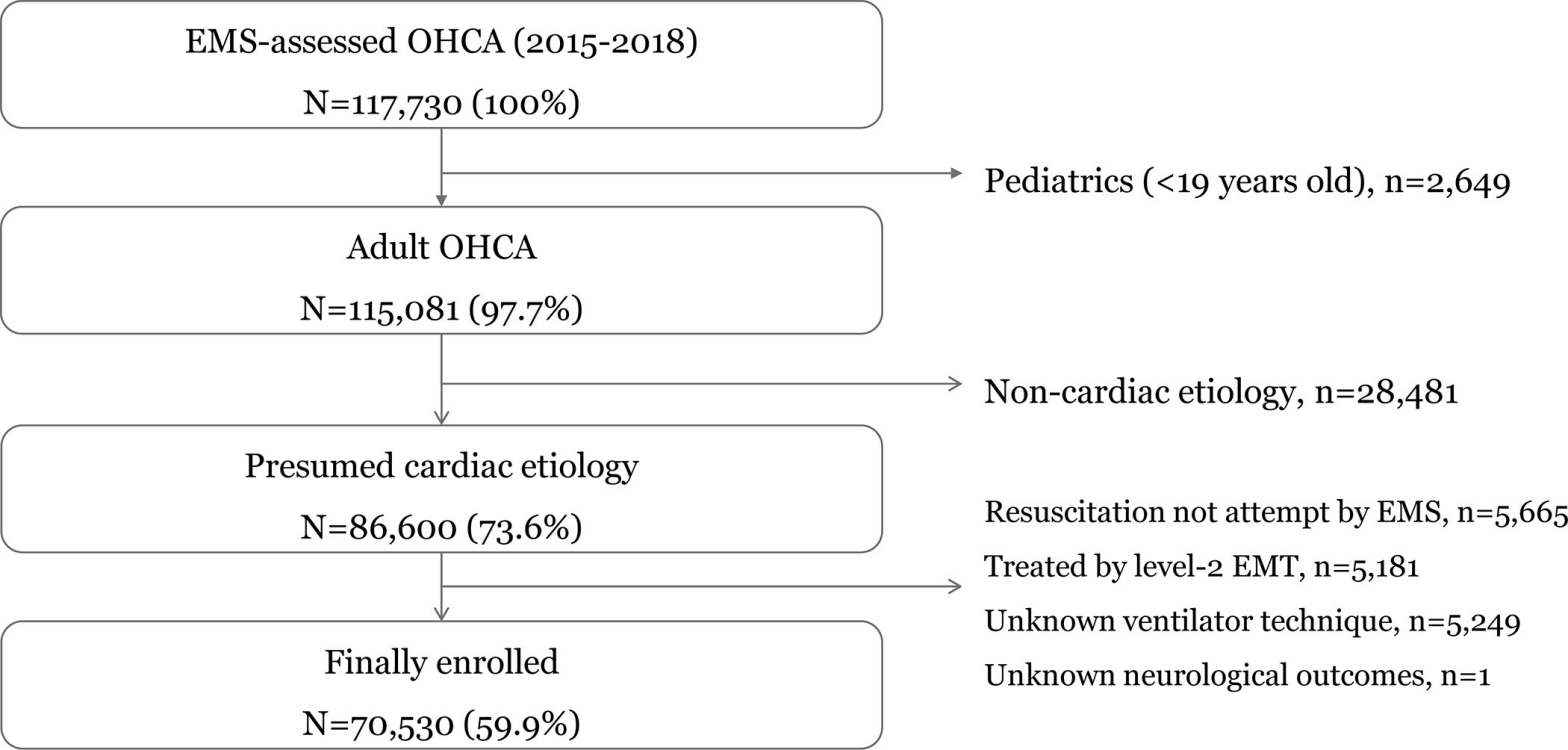
Study populations of observational study.

Among 117,730 EMS-assessed OHCA cases that occurred within the study period, 70,530 (59.9%) met the inclusion criteria and were further evaluated (Fig 1).

We excluded patients who were <19 years old (n = 2,649), had non-cardiac etiology (n = 28,481), did not receive resuscitation efforts by EMS providers (n = 5,665), were treated by level-2 EMTs (n = 5,181), and had missing records of the airway technique used and the neurological outcomes (n = 5,250).

The demographic characteristics of patients with OHCA, based on the Utstein elements, according to the airway management technique used are summarized in Table 1. Of the 70,530 eligible patients, BVM, SGA and ETI were used in 26,547 (37.6%), 38,391 (54.4%), and 5,592 (7.9%) patients, respectively. The overall prehospital ROSC, survival to discharge, and favorable survival to discharge rates were 36.2%, 9.3%, and 5.8% respectively. During the study period, the use of advanced airway procedures such as SGA and ETI increased over the years. Patients in the ETI and SGA groups were more likely to be from the metropolitan areas, have a witnessed arrest, received CPR from a bystander, had initial shockable rhythms on the ECG, and had more number of EMTs who attended to them. Scene time interval was longer and the use of mechanical chest compression device and epinephrine was higher in the ETI and SGA groups. There were a total of 1,198 (21.4%) survival to discharge patients in the ETI group, 8,590 (22.4%) in SGA group, and 5,537 (20.9%) in BVM group (p < 0.01). There was no significant difference in the neurological recovery across the airway intervention techniques.

**Table 1:** Characteristics of out-of-hospital cardiac arrest patients by airway management techniques.

Variables		All	Pre-hospital airway management			
			BVM	SGA	ETI	P-value
		N (%)	N (%)	N (%)	N (%)	
All		70,530 (100.0)	26,547 (100.0)	38,391 (100.0)	5,592 (100.0)	
Year						<0.01
	2015	15,659 (22.2)	10,261 (38.7)	4,370 (11.4)	1,028 (18.4)	
	2016	17,159 (24.3)	7,702 (29.0)	8,114 (21.1)	1,343 (24.0)	
	2017	17,766 (25.2)	5,640 (21.2)	10,705 (27.9)	1,421 (25.4)	
	2018	19,946 (28.3)	2,944 (11.1)	15,202 (39.6)	1,800 (32.2)	
Age						<0.01
	18–65	24,392 (34.6)	8,894 (33.5)	13,648 (35.5)	1,850 (33.1)	
	65-	46,138 (65.4)	17,653 (66.5)	24,743 (64.5)	3,742 (66.9)	
Gender						<0.01
	Male	44,782 (63.5)	16,392 (61.7)	24,784 (64.6)	3,606 (64.5)	
	Female	25,748 (36.5)	10,155 (38.3)	13,607 (35.4)	1,986 (35.5)	
Metropolis						<0.01
	Yes	15,791 (22.4)	5,455 (20.5)	9,058 (23.6)	1,278 (22.9)	
	No	54,739 (77.6)	21,092 (79.5)	29,333 (76.4)	4,314 (77.1)	
Diabetes						<0.01
	Yes	23,977 (34.0)	8,350 (31.5)	13,678 (35.6)	1,949 (34.9)	
	No	46,553 (66.0)	18,197 (68.5)	24,713 (64.4)	3,643 (65.1)	
Hypertension						<0.01
	Yes	11,951 (16.9)	4,015 (15.1)	7,009 (18.3)	927 (16.6)	
	No	58,579 (83.1)	22,532 (84.9)	31,382 (81.7)	4,665 (83.4)	
Heart disease						<0.01
	Yes	30,824 (43.7)	10,048 (37.8)	17,345 (45.2)	3,431 (61.4)	
	No	39,706 (56.3)	16,499 (62.2)	21,046 (54.8)	2,161 (38.6)	
Place						<0.01
	Private	53,521 (75.9)	19,588 (73.8)	29,458 (76.7)	4,475 (80.0)	
	Public	17,009 (24.1)	6,959 (26.2)	8,933 (23.3)	1,117 (20.0)	
Witness						<0.01
	Yes	35,955 (51.0)	13,142 (49.5)	19,903 (51.8)	2,910 (52.0)	
	No	34,575 (49.0)	13,405 (50.5)	18,488 (48.2)	2,682 (48.0)	
Bystander CPR						<0.01
	Yes	14,705 (20.8)	4,209 (15.9)	9,124 (23.8)	1,372 (24.5)	
	No	55,825 (79.2)	22,338 (84.1)	29,267 (76.2)	4,220 (75.5)	
Initial ECG rhythm						<0.01
	Shockable	12,668 (18.0)	4,253 (16.0)	7,351 (19.1)	1,064 (19.0)	
	Non-shockable	57,862 (82.0)	22,294 (84.0)	31,040 (80.9)	4,528 (81.0)	
Response time interval						<0.01
	0~5 min	21,212 (30.1)	7,918 (29.8)	11,274 (29.4)	2,020 (36.1)	
	6~10 min	36,569 (51.8)	13,232 (49.8)	20,529 (53.5)	2,808 (50.2)	
	11~ min	12,749 (18.1)	5,397 (20.3)	6,588 (17.2)	764 (13.7)	
Scene time interval						<0.01
	0~8 min	13,033 (18.5)	8,238 (31.0)	4,368 (11.4)	427 (7.6)	
	9~16 min	37,085 (52.6)	14,471 (54.5)	19,745 (51.4)	2,869 (51.3)	
	16~ min	20,412 (28.9)	3,838 (14.5)	14,278 (37.2)	2,296 (41.1)	
Transport time interval						<0.01
	0~4 min	21,216 (30.1)	7,861 (29.6)	11,687 (30.4)	1,668 (29.8)	
	5~8 min	26,056 (36.9)	9,359 (35.3)	14,521 (37.8)	2,176 (38.9)	
	9~ min	23,258 (33.0)	9,327 (35.1)	12,183 (31.7)	1,748 (31.3)	
EMT number						<0.01
	3 persons	32,894 (46.6)	9,308 (35.1)	20,115 (52.4)	3,471 (62.1)	
	1 or 2 persons	37,636 (53.4)	17,239 (64.9)	18,276 (47.6)	2,121 (37.9)	
Mechanical CPR						<0.01
	Yes	5,955 (8.4)	782 (2.9)	3,815 (9.9)	1,358 (24.3)	
	No	64,575 (91.6)	25,765 (97.1)	34,576 (90.1)	4,234 (75.7)	
EMS defibrillation						<0.01
	Yes	17,384 (24.6)	6,003 (22.6)	9,947 (25.9)	1,434 (25.6)	
	No	53,146 (75.4)	20,544 (77.4)	28,444 (74.1)	4,158 (74.4)	
EMS epinephrine						<0.01
	Yes	9,716 (13.8)	1,060 (4.0)	7,647 (19.9)	1,009 (18.0)	
	No	60,814 (86.2)	25,487 (96.0)	30,744 (80.1)	4,583 (82.0)	
ED level						<0.01
	Level 1	13,158 (18.7)	3,690 (13.9)	8,349 (21.7)	1,119 (20.0)	
	Level 2	34,075 (48.3)	11,981 (45.1)	19,127 (49.8)	2,967 (53.1)	
	Level 3	23,297 (33.0)	10,876 (41.0)	10,915 (28.4)	1,506 (26.9)	
TTM						<0.01
	Yes	68,278 (96.8)	25,949 (97.7)	36,954 (96.3)	5,375 (96.1)	
	No	2,252 (3.2)	598 (2.3)	1,437 (3.7)	217 (3.9)	
Reperfusion						<0.01
	Yes	4,328 (6.1)	1,214 (4.6)	2,758 (7.2)	356 (6.4)	
	No	66,202 (93.9)	25,333 (95.4)	35,633 (92.8)	5,236 (93.6)	
ECMO						<0.01
	Yes	730 (1.0)	202 (0.8)	458 (1.2)	70 (1.3)	
	No	69,800 (99.0)	26,345 (99.2)	37,933 (98.8)	5,522 (98.7)	
Study outcomes						
	Prehospital ROSC	25,510 (36.2)	8,842 (33.3)	14,533 (37.9)	2,135 (38.2)	<0.01
	Survival to discharge	6,565 (9.3)	2,439 (9.2)	3,618 (9.4)	508 (9.1)	0.5
	Good CPC	4,102 (5.8)	1,540 (5.8)	2,262 (5.9)	300 (5.4)	0.29

ETI, endo-tracheal intubation; SGA, supra-glottic airway; BVM, bag-valve mask; CPR, cardiopulmonary resuscitation; ECG, electrocardiogram; EMT, emergency medical technician; EMS, emergency medical service; ED, emergency department; TTM, targeted temperature management; ECMO, extracorporeal membrane oxygenation; ROSC, return of spontaneous circulation; CPC, cerebral performance category.

The demographics of OHCA patients by TTI are summarized in Table 2.

**Table 2:** Characteristics of out-of-hospital cardiac arrest patients by transport time interval.

Variables			All	Transport time interval			
				0-4min	4–8 min	8min-	P-value
			N (%)	N (%)	N (%)	N (%)	
All			70,530 (100.0)	21,216 (100.0)	26,056 (100.0)	23,258 (100.0)	
Year							<0.01
	2015		15,659 (22.2)	4,626 (21.8)	6,086 (23.4)	4,947 (21.3)	
	2016		17,159 (24.3)	5,109 (24.1)	6,407 (24.6)	5,643 (24.3)	
	2017		17,766 (25.2)	5,472 (25.8)	6,425 (24.7)	5,869 (25.2)	
	2018		19,946 (28.3)	6,009 (28.3)	7,138 (27.4)	6,799 (29.2)	
Age							<0.01
	18–65		24,392 (34.6)	7,554 (35.6)	9,072 (34.8)	7,766 (33.4)	
	65-		46,138 (65.4)	13,662 (64.4)	16,984 (65.2)	15,492 (66.6)	0.77
Gender							<0.01
	Male		44,782 (63.5)	13,454 (63.4)	16,517 (63.4)	14,811 (63.7)	
	Female		25,748 (36.5)	7,762 (36.6)	9,539 (36.6)	8,447 (36.3)	
Metropolis							<0.01
	Yes		15,791 (22.4)	4,802 (22.6)	6,051 (23.2)	4,938 (21.2)	
	No		54,739 (77.6)	16,414 (77.4)	20,005 (76.8)	18,320 (78.8)	
Diabetes							<0.01
	Yes		23,977 (34.0)	7,158 (33.7)	9,159 (35.2)	7,660 (32.9)	
	No		46,553 (66.0)	14,058 (66.3)	16,897 (64.8)	15,598 (67.1)	
Hypertension							<0.01
	Yes		11,951 (16.9)	3,635 (17.1)	4,437 (17.0)	3,879 (16.7)	
	No		58,579 (83.1)	17,581 (82.9)	21,619 (83.0)	19,379 (83.3)	
Heart disease							0.4
	Yes		30,824 (43.7)	9,938 (46.8)	13,511 (51.9)	7,375 (31.7)	
	No		39,706 (56.3)	11,278 (53.2)	12,545 (48.1)	15,883 (68.3)	
Place							<0.01
	Private		53,521 (75.9)	16,119 (76.0)	20,243 (77.7)	17,159 (73.8)	
	Public		17,009 (24.1)	5,097 (24.0)	5,813 (22.3)	6,099 (26.2)	
Witness							<0.01
	Yes		35,955 (51.0)	10,430 (49.2)	13,191 (50.6)	12,334 (53.0)	
	No		34,575 (49.0)	10,786 (50.8)	12,865 (49.4)	10,924 (47.0)	
Bystander CPR							<0.01
	Yes		14,705 (20.8)	4,062 (19.1)	5,797 (22.2)	4,846 (20.8)	
	No		55,825 (79.2)	17,154 (80.9)	20,259 (77.8)	18,412 (79.2)	
Initial ECG rhythm							<0.01
	Shockable		12,668 (18.0)	3,769 (17.8)	4,540 (17.4)	4,359 (18.7)	
	Non-shockable		57,862 (82.0)	17,447 (82.2)	21,516 (82.6)	18,899 (81.3)	
Response time interval							<0.01
	0~5 min		21,212 (30.1)	8,606 (40.6)	7,742 (29.7)	4,864 (20.9)	
	6~10 min		36,569 (51.8)	11,016 (51.9)	14,890 (57.1)	10,663 (45.8)	
	11~ min		12,749 (18.1)	1,594 (7.5)	3,424 (13.1)	7,731 (33.2)	
Scene time interval							<0.01
	0~8 min		13,033 (18.5)	3,443 (16.2)	4,431 (17.0)	5,159 (22.2)	
	9~16 min		37,085 (52.6)	11,234 (53.0)	14,102 (54.1)	11,749 (50.5)	
	16~ min		20,412 (28.9)	6,539 (30.8)	7,523 (28.9)	6,350 (27.3)	
EMT number							<0.01
	3 persons		32,894 (46.6)	10,485 (49.4)	13,681 (52.5)	8,728 (37.5)	
	1 or 2 persons		37,636 (53.4)	10,731 (50.6)	12,375 (47.5)	14,530 (62.5)	
Prehospital airway							
	BVM		26,547 (37.6)	7,861 (37.1)	9,359 (35.9)	9,327 (40.1)	
	SGA		38,391 (54.4)	11,687 (55.1)	14,521 (55.7)	12,183 (52.4)	
	ETI		5,592 (7.9)	1,668 (7.9)	2,176 (8.4)	1,748 (7.5)	
Mechanical CPR							<0.01
	Yes		5,955 (8.4)	1,572 (7.4)	2,467 (9.5)	1,916 (8.2)	
	No		64,575 (91.6)	19,644 (92.6)	23,589 (90.5)	21,342 (91.8)	
EMS defibrillation							<0.01
	Yes		17,384 (24.6)	4,954 (23.4)	6,125 (23.5)	6,305 (27.1)	
	No		53,146 (75.4)	16,262 (76.6)	19,931 (76.5)	16,953 (72.9)	
EMS epinephrine							0.02
	Yes		9,716 (13.8)	3,031 (14.3)	3,575 (13.7)	3,110 (13.4)	
	No		60,814 (86.2)	18,185 (85.7)	22,481 (86.3)	20,148 (86.6)	
ED level							<0.01
	Level 1		13,158 (18.7)	3,301 (15.6)	5,090 (19.5)	4,767 (20.5)	
	Level 2		34,075 (48.3)	9,936 (46.8)	13,483 (51.7)	10,656 (45.8)	
	Level 3		23,297 (33.0)	7,979 (37.6)	7,483 (28.7)	7,835 (33.7)	
TTM							<0.01
	Yes		68,278 (96.8)	20,608 (97.1)	25,119 (96.4)	22,551 (97.0)	
	No		2,252 (3.2)	608 (2.9)	937 (3.6)	707 (3.0)	
Reperfusion							<0.01
	Yes		4,328 (6.1)	1,201 (5.7)	1,643 (6.3)	1,484 (6.4)	
	No		66,202 (93.9)	20,015 (94.3)	24,413 (93.7)	21,774 (93.6)	
ECMO							<0.01
	Yes		730 (1.0)	201 (0.9)	314 (1.2)	215 (0.9)	
	No		69,800 (99.0)	21,015 (99.1)	25,742 (98.8)	23,043 (99.1)	
Study outcomes							
	Prehospital ROSC		25,510 (36.2)	7,881 (37.1)	9,675 (37.1)	7,954 (34.2)	<0.01
	Survival to discharge		6,565 (9.3)	1,948 (9.2)	2,426 (9.3)	2,191 (9.4)	0.69
	Good CPC		4,102 (5.8)	1,182 (5.6)	1,467 (5.6)	1,453 (6.2)	<0.01

CPR, cardiopulmonary resuscitation; ECG, electrocardiogram; BVM, bag-valve mask; SGA, supraglottic airway; ETI, endotracheal intubation; EMS, emergency medical service; ED, emergency department; TTM, targeted temperature management; ECMO, extracorporeal membrane oxygenation; ROSC, return of spontaneous circulation; CPC, cerebral performance category.

Patients with longer TTI (more than 8 minutes) were more likely to be from the non-metropolitan areas, had initial shockable rhythms in ECG, had fewer number of EMT members who attended to them, and were likely to be transported to level-1 ED. The survival to discharge was lower in patients with longer TTI, while the neurological recovery was better.

### Main results

A comparison of the study outcomes is presented in Table 3. After adjusting for possible confounders, patients in the SGA and ETI groups had a significantly higher likelihood of survival to discharge than those in the BVM group, (adjusted OR, 1.07; 95% CI, [1.01–1.12] and 1.11 [1.03–1.20]).

**Table 3:** Multivariable adjusted logistic regression analysis model for outcomes by airway management technique.

Airway management		Total	Outcomes		Model 1			Model 2			
		N	n	%	AOR	95% CI		AOR	95% CI		
Survival to discharge											
	Total	70530	6565	9.3							
	BVM (reference)	26547	2439	9.2	1.00			1.00			
	SGA	38391	3618	9.4	0.96	0.92	1.01	1.07	1.01		1.12
	ETI	5592	508	9.1	0.91	0.85	0.98	1.11	1.03		1.20
Good CPC											
	Total	70530	4102	5.8							
	BVM (reference)	26547	1540	5.8	1.00			1.00			
	SGA	38391	2262	5.9	0.80	0.80	0.86	0.85	0.77		0.93
	ETI	5592	300	5.4	0.76	0.76	0.87	0.94	0.80		1.08

AOR, adjusted odds ratio; CI, confidence interval; BVM, bag-valve mask; SGA, supraglottic airway; ETI, endotracheal intubation; CPR, cardiopulmonary resuscitation, ECG, electrocardiogram; EMT, emergency medical technician; EMS, emergency medical service; ED, emergency department.

Model 1: adjusted for year of arrest, age, and gender.

Model 2: model 1 + metropolis, diabetes, hypertension, heart disease, private, witness, bystander CPR, initial ECG rhythm, transport time interval, EMT number, mechanical CPR, EMS defibrillation.

In the fully adjusted model (Model 2), the odds of neurologically favorable outcomes (Good CPC) were significantly lower in the SGA group than in the BVM group (adjusted OR, 0.85 [0.77–0.93]), whereas, it was statistically non-significant in the ETI group compared to that in the BVM group (adjusted OR, 0.94 (0.80–1.08)).

### Interaction analysis

After adjusting for the other covariables in the interaction model, the adjusted odd ratios (AOR) of the study outcomes differed across the prehospital airway interventions according to the TTI (Table 4).

**Table 4:** Interaction analysis for outcomes of prehospital airway management technique according to transport time interval.

			Survival to discharge				Good CPC			
			AOR	95% CI		P-value	AOR	95% CI		P-value
Transport time interval	0~4 min	BVM	1.00			0.01	1.00			0.47
		SGA	1.04	0.96	1.13		0.89	0.77	1.04	
		ETI	1.01	0.87	1.16		1.09	0.83	1.43	
	4~8min	BVM	1.00				1.00			
		SGA	1.08	1.01	1.17		0.82	0.71	0.93	
		ETI	1.06	0.93	1.21		0.76	0.58	0.95	
	8min~	BVM	1.00				1.00			
		SGA	1.18	1.09	1.28		0.95	0.80	1.10	
		ETI	1.39	1.21	1.60		0.91	0.70	1.12	

AOR, adjusted odds ratio; CI, confidence interval; CPC, cerebral performance category; BVM, bag-valve mask; SGA, supraglottic airway; ETI, endotracheal intubation; min, minutes.

For survival to discharge with BVM as reference, the rates of survival to discharge with SGA and ETI were significantly higher only in groups with TTI more than 8 minutes (1.18 (1.09–1.28), 1.39 (1.21–1.60)).

## Discussion

In our study, the survival to discharge rates were higher among the patients who received advanced airway management (ETI and SGA) compared with those who received BVM, and the differences in outcomes were more prominent in patients with longer TTI (TTI more than 8 minutes). However, the neurologically favorable survival to discharge in the SGA group was lower than that in the BVM group, while there was no significant difference between the ETI and BVM groups.

The results of our study indicating that AAM has a higher rate of survival to discharge than BVM are somewhat different from those of previous studies. In a network meta-analysis comparing the clinical outcomes between BVM, SGA and ETI, there were no differences in the survival to discharge or good neurological recovery rates between these airway interventions [16]. In the Cardiac Arrest Registry to Enhance Survival (CARES) of US and Hasegawa’s study of a Japanese nationwide cohort of patients with OHCA, the survival outcomes were higher among patients who did not receive AAM than in those who received AAM [17, 18]. In several observational studies, including the two studies above, it was suggested that prehospital AAM does not improve the survival and neurological outcomes, but might lead to a decreased rate of favorable neurological outcomes compared to those with BVM. Only a few studies have demonstrated the benefits from ETI or SGA. Contrary to the results of our study, in the 3 RCTs published recently [2–4], the ETI or SGA did not show better clinical outcomes than the BVM. Although, our study has limitation as an observational study, it showed better clinical outcome compared to BVM, and suggested the possibility that the longer TTI, the more useful the AAM in the ‘scoop and run’ system.

Advanced airway is a definitive airway management technique that greatly enhances gas exchange and allows continued chest compressions once it is successfully performed [19]. However, it has also been well documented that prehospital ETI is a complex psychomotor task and that the EMT personnel experience difficulty in gaining and maintaining competency in this skill. Moreover, advanced airway devices might impinge on the vascular structures. Kim et al. observed that SGA was associated with decreasing carotid blood flow during CPR in a porcine model [11]. Moreover, patients who received AAM during CPR might not have achieved ROSC before receiving AAM, a phenomenon now known as the “resuscitation time bias” that would show better outcomes in the no AAM group [20]. Despite the potential of ‘resuscitation time bias, our study still found positive effect of advance airway on survival to discharge, especially longer transport time interval.

Several studies, including one meta-analysis, have reported that a longer TTI is not associated with decreased survival [9, 10, 16]. However, Park et al. [21] reported a longer TTI had a negative effect on the neurological outcome in OHCA patients without prehospital ROSC, by analyzing the national OHCA registry of Korea. Considering characteristics of airway management and ‘scoop and run’ EMS system of Korea, it is assumed that the transport time interval may affect the clinical outcomes of airway management technique, although it was not considered in the previous RCT and meta-analysis. In our study, the survival outcome with AAM was better when the TTI was more than 8 minutes compared to that in the group in which it was under 4 minutes. Regarding the initiation of airway management, there are several reports that correct insertion of AAM devices is difficult and consumes more time than BVM, consequently leading to an increase in the no flow time, which worsens the survival outcome [19, 22]. Several recent studies demonstrated that there is inadequate evidence to show a difference in survival and neurologic outcome with the use of BVM compared with AAM [17, 18]. However, after starting the transportation, the longer the TTI, the more difficult it is to maintain the mask sealing of BVM, and the ventilation accuracy decreases [12]. Conversely, once AAM is initiated, it can supply more stable ventilation compared to BVM during transport. Hence, it is thought that longer the TTI, higher are the odds for survival to discharge of OHCA patients with AAM.

Good neurological recovery, which was the secondary outcome of our study, improved with an increase in TTI. This somewhat paradoxical result may be originated that emphasizing on-scene resuscitation in Korea after year of 2017, and the transfer to level-1 ED where post-cardiac arrest care is possible after on-scene resuscitation, however, further research is needed.

In summary, our study showed that AAM increases the rate of survival to discharge compared to BVM, and this difference was more pronounced with an increase in the TTI. BVM should be preferred for short-distance transportation; however, according to our study, AAM should be considered for long-distance transportation.

## Limitations

This study has several limitations. First, the Korean nationwide EMS-assessed OHCA patient database is not designed for collecting information on the airway management technique. Therefore, our cohort lacks details about airway management, such as the number and duration of airway insertion attempts, failed airway insertion attempts, and the proficiency or experience of the EMS personnel. It was also unknown whether advanced airway intervention was performed during CPR or after ROSC. Second, the quality of CPR is a strong prognostic factor. The association between the prehospital airway management intervention and clinical outcomes might have been confounded by the quality of CPR. However, the CPR quality was not captured in our database. Third, we could not include data about the complications associated with airway management technique. fourth, while we used multivariable analysis, unmeasured and unmeasurable confounders might have influenced the clinical outcomes in this study. fifth, we excluded 5,665, patients without information of airway management technique, which is our main exposure from our analysis. It is possible that these excluded patients may have affected the study outcomes. Lastly, the reference for classifying TTI based on 8 minutes is not sufficient. it was established because the goal of TTI of OHCA patients in Korea is 8 minutes and becomes the standard when patients are divided into tertiles.

## Conclusions

In this Korean population-based study of an OHCA cohort, the survival to discharge was significantly higher among patients who received ETI and SGA than in those who received BVM. The transport time interval influenced the effect of prehospital airway management on the clinical outcomes after OHCA. These results emphasize that when EMS providers select an airway management technique, the expected TTI should be considered.

## References

[1] Berdowski J, Berg RA, Tijssen JG, Koster RW. Global incidences of out-of-hospital cardiac arrest and survival rates: Systematic review of 67 prospective studies. Resuscitation. 2010;81(11):1479–87. doi: 10.1016/j.resuscitation.2010.08.006 20828914

[2] Benger JR, Kirby K, Black S, Brett SJ, Clout M, Lazaroo MJ, et al. Effect of a strategy of a supraglottic airway device vs tracheal intubation during out-of-hospital cardiac arrest on functional outcome: the AIRWAYS-2 randomized clinical trial. Jama. 2018;320(8):779–91. doi: 10.1001/jama.2018.11597 30167701 PMC6142999

[3] Jabre P, Penaloza A, Pinero D, Duchateau FX, Borron SW, Javaudin F, et al. Effect of Bag-Mask Ventilation vs Endotracheal Intubation During Cardiopulmonary Resuscitation on Neurological Outcome After Out-of-Hospital Cardiorespiratory Arrest: A Randomized Clinical Trial. Jama. 2018;319(8):779–87. doi: 10.1001/jama.2018.0156 29486039 PMC5838565

[4] Wang HE, Schmicker RH, Daya MR, Stephens SW, Idris AH, Carlson JN, et al. Effect of a strategy of initial laryngeal tube insertion vs endotracheal intubation on 72-hour survival in adults with out-of-hospital cardiac arrest: a randomized clinical trial. Jama. 2018;320(8):769–78. doi: 10.1001/jama.2018.7044 30167699 PMC6583103

[5] Jentzer JC, Clements CM, Wright RS, White RD, Jaffe AS. Improving Survival From Cardiac Arrest: A Review of Contemporary Practice and Challenges. Ann Emerg Med. 2016;68(6):678–89. doi: 10.1016/j.annemergmed.2016.05.022 27318408

[6] Myat A, Song KJ, Rea T. Out-of-hospital cardiac arrest: current concepts. Lancet. 2018;391(10124):970–9. doi: 10.1016/S0140-6736(18)30472-0 29536861

[7] Lyon RM, Ferris JD, Young DM, McKeown DW, Oglesby AJ, Robertson C. Field intubation of cardiac arrest patients: a dying art? Emerg Med J. 2010;27(4):321–3. doi: 10.1136/emj.2009.076737 20385694

[8] Wang HE, Benger JR. Endotracheal intubation during out-of-hospital cardiac arrest: New insights from recent clinical trials. J Am Coll Emerg Physicians Open. 2019;1(1):24–9. doi: 10.1002/emp2.12003 33000010 PMC7493580

[9] Aufderheide TP, Lurie KG. Death by hyperventilation: a common and life-threatening problem during cardiopulmonary resuscitation. Critical care medicine. 2004;32(9 Suppl):S345–51. doi: 10.1097/01.ccm.0000134335.46859.09 15508657

[10] Wang HE, Simeone SJ, Weaver MD, Callaway CW. Interruptions in cardiopulmonary resuscitation from paramedic endotracheal intubation. Annals of emergency medicine. 2009;54(5):645–52.e1. doi: 10.1016/j.annemergmed.2009.05.024 19573949

[11] Kim TH, Hong KJ, Shin SD, Lee JC, Choi DS, Chang I, et al. Effect of endotracheal intubation and supraglottic airway device placement during cardiopulmonary resuscitation on carotid blood flow over resuscitation time: An experimental porcine cardiac arrest study. Resuscitation. 2019;139:269–74. doi: 10.1016/j.resuscitation.2019.04.020 31009692

[12] Odegaard S, Pillgram M, Berg NE, Olasveengen T, Kramer-Johansen J. Time used for ventilation in two-rescuer CPR with a bag-valve-mask device during out-of-hospital cardiac arrest. Resuscitation. 2008;77(1):57–62. doi: 10.1016/j.resuscitation.2007.11.005 18164533

[13] Jung E, Lee SY, Park JH, Ro YS, Hong KJ, Song KJ, et al. Interaction Effects Between Targeted Temperature Management and Hypertension on Survival Outcomes After Out-of-Hospital Cardiac Arrest: A National Observational Study from 2009 to 2016. Therapeutic hypothermia and temperature management. 2020;10(3):141–7. doi: 10.1089/ther.2019.0015 31414970

[14] Jung E, Park JH, Lee SY, Ro YS, Hong KJ, Song KJ, et al. Mechanical Chest Compression Device for Out-Of-Hospital Cardiac Arrest: A Nationwide Observational Study. The Journal of emergency medicine. 2020;58(3):424–31. doi: 10.1016/j.jemermed.2019.11.022 32178958

[15] Perkins GD, Jacobs IG, Nadkarni VM, Berg RA, Bhanji F, Biarent D, et al. Cardiac arrest and cardiopulmonary resuscitation outcome reports: update of the Utstein Resuscitation Registry Templates for Out-of-Hospital Cardiac Arrest: a statement for healthcare professionals from a task force of the International Liaison Committee on Resuscitation (American Heart Association, European Resuscitation Council, Australian and New Zealand Council on Resuscitation, Heart and Stroke Foundation of Canada, InterAmerican Heart Foundation, Resuscitation Council of Southern Africa, Resuscitation Council of Asia); and the American Heart Association Emergency Cardiovascular Care Committee and the Council on Cardiopulmonary, Critical Care, Perioperative and Resuscitation. Circulation. 2015;132(13):1286–300. doi: 10.1161/CIR.0000000000000144 25391522

[16] Cha WC, Lee SC, Shin SD, Song KJ, Sung AJ, Hwang SS. Regionalisation of out-of-hospital cardiac arrest care for patients without prehospital return of spontaneous circulation. Resuscitation. 2012;83(11):1338–42. doi: 10.1016/j.resuscitation.2012.03.024 22446564

[17] Hasegawa K, Hiraide A, Chang Y, Brown DF. Association of prehospital advanced airway management with neurologic outcome and survival in patients with out-of-hospital cardiac arrest. Jama. 2013;309(3):257–66. doi: 10.1001/jama.2012.187612 23321764

[18] McMullan J, Gerecht R, Bonomo J, Robb R, McNally B, Donnelly J, et al. Airway management and out-of-hospital cardiac arrest outcome in the CARES registry. Resuscitation. 2014;85(5):617–22. doi: 10.1016/j.resuscitation.2014.02.007 24561079

[19] Benoit JL, Prince DK, Wang HE. Mechanisms linking advanced airway management and cardiac arrest outcomes. Resuscitation. 2015;93:124–7. doi: 10.1016/j.resuscitation.2015.06.005 26073275 PMC4506864

[20] Andersen LW, Grossestreuer AV, Donnino MW. "Resuscitation time bias"-A unique challenge for observational cardiac arrest research. Resuscitation. 2018;125:79–82. doi: 10.1016/j.resuscitation.2018.02.006 29425975 PMC6080954

[21] Park JH, Kim YJ, Ro YS, Kim S, Cha WC, Shin SD. The Effect of Transport Time Interval on Neurological Recovery after Out-of-Hospital Cardiac Arrest in Patients without a Prehospital Return of Spontaneous Circulation. Journal of Korean medical science. 2019;34(9):e73. doi: 10.3346/jkms.2019.34.e73 30863269 PMC6406038

[22] Penketh JA, Nolan JP, Skrifvars MB, Rylander C, Frenell I, Tirkkonen J, et al. Airway management during in-hospital cardiac arrest: An international, multicentre, retrospective, observational cohort study. Resuscitation. 2020;153:143–8. doi: 10.1016/j.resuscitation.2020.05.028 32479867

